# Country-level social mobility and inequalities in adolescent health behaviours in 32 countries

**DOI:** 10.1093/eurpub/ckac129.053

**Published:** 2022-10-25

**Authors:** H Schmengler, M Peeters, GWJM Stevens, AE Kunst, K Delaruelle, M Dierckens, L Charrier, D Weinberg, AJ Oldehinkel, WAM Vollebergh

**Affiliations:** 1 Department of Interdisciplinary Social Science, Utrecht University, Utrecht, Netherlands; 2 Department of Public and Occupational Health, Amsterdam UMC, Amsterdam, Netherlands; 3 Department of Public Health and Primary Care, Ghent University, Ghent, Belgium; 4 Department of Sociology, Ghent University, Ghent, Belgium; 5 Department of Public Health and Paediatrics, University of Torino, Turin, Italy; 6 Department of Psychiatry, University Medical Center of Groningen, Groningen, Netherlands

## Abstract

**Background:**

Higher family affluence is associated with healthier behaviours in adolescents, but the strength of this association varies across countries. Differences in social mobility at the country-level, i.e. the extent to which adolescents develop a different socioeconomic status (SES) than their parents, may partially explain why the association between family affluence and adolescent health behaviours is stronger in some countries than in others.

**Methods:**

Using data from adolescents aged 11-15 years from 32 different countries, participating in the 2017/2018 wave of the Health Behaviour in School-aged Children (HBSC) study (N = 185,086), we employed multilevel regression models with cross-level interactions to examine whether country-level social mobility moderates the association between family affluence and adolescent health behaviours (i.e. moderate-to-vigorous physical activity, vigorous physical activity, healthy foods consumed, unhealthy foods consumed, having breakfast regularly, weekly smoking).

**Results:**

Higher family affluence was more strongly associated with higher levels of physical activity in countries characterized by high levels of social mobility (cross-level interaction linear regression coefficient 0.34; 95% CI 0.08 to 0.60; p = 0.009 for moderate-to-vigorous physical activity, and 0.31; 0.11 to 0.50; p = 0.002 for vigorous physical activity). No cross-level interactions were found for any of the other health behaviours.

**Conclusions:**

Our findings suggest that differences in social mobility at the country-level may contribute to cross-national variations in socioeconomic inequalities in adolescent physical activity. Further research can shed light on the mechanisms linking country-level social mobility to inequalities in adolescent physical activity to identify targets for policy and interventions.

**Key messages:**

• This is one of the first studies to investigate country-level social mobility in relation to health equity. Inequalities in adolescent physical activity were steeper in socially mobile countries.

• Stronger efforts to engage adolescents from low-affluent families in physical activity may be necessary in countries characterized by high levels of social mobility.

